# The Fragile Breakage versus Random Breakage Models of Chromosome Evolution

**DOI:** 10.1371/journal.pcbi.0020014

**Published:** 2006-02-24

**Authors:** Qian Peng, Pavel A Pevzner, Glenn Tesler

**Affiliations:** 1 Department of Computer Science and Engineering, University of California San Diego, La Jolla, California, United States of America; 2 Department of Mathematics, University of California San Diego, La Jolla, California, United States of America; University of California San Diego, United States of America

## Abstract

For many years, studies of chromosome evolution were dominated by the random breakage theory, which implies that there are no rearrangement hot spots in the human genome. In 2003, Pevzner and Tesler argued against the random breakage model and proposed an alternative “fragile breakage” model of chromosome evolution. In 2004, Sankoff and Trinh argued against the fragile breakage model and raised doubts that Pevzner and Tesler provided any evidence of rearrangement hot spots. We investigate whether Sankoff and Trinh indeed revealed a flaw in the arguments of Pevzner and Tesler. We show that Sankoff and Trinh's synteny block identification algorithm makes erroneous identifications even in small toy examples and that their parameters do not reflect the realities of the comparative genomic architecture of human and mouse. We further argue that if Sankoff and Trinh had fixed these problems, their arguments in support of the random breakage model would disappear. Finally, we study the link between rearrangements and regulatory regions and argue that long regulatory regions and inhomogeneity of gene distribution in mammalian genomes may be responsible for the breakpoint reuse phenomenon.

## Synopsis

Genomes are constantly changing. If a genome is compared to a continental landform, then one type of change—point mutations—is analogous to gradual changes in the landscape due to erosion by wind and water. A second type of change—genome rearrangements—comprises evolutionary “earthquakes” that dramatically change the landscape. A fundamental question in studies of chromosome evolution is whether these earthquakes are happening along evolutionary “faults” (hot spots of rearrangements) or at “random” genomic positions. For many years, studies of chromosome evolution were dominated by the random breakage theory, which implies that there are no rearrangement hot spots in the human genome. In 2003, Pevzner and Tesler argued against the random breakage model and proposed an alternative “fragile breakage” model of chromosome evolution. In 2004, Sankoff and Trinh performed a series of computational simulations that argued against the fragile breakage model and raised doubts that Pevzner and Tesler provided any evidence of rearrangement hot spots. The authors show that Sankoff and Trinh's simulation misidentifies synteny blocks, that it does not accurately simulate what Pevzner and Tesler (2003) did, and that the parameters of Sankoff and Trinh do not reflect the realities of the comparative genomic architecture of human and mouse.

## Introduction

Genomes are constantly changing. If a genome is compared to a continental landform, then one type of change—point mutations—is analogous to gradual changes in the landscape due to erosion by wind and water. A second type of change—genome rearrangements—comprises evolutionary “earthquakes” that dramatically change the landscape. A fundamental question in studies of chromosome evolution is whether these earthquakes are happening along evolutionary “faults” (hot spots of rearrangements) or at “random” genomic positions.

In a landmark paper in 1984, Nadeau and Taylor [1] estimated that there are approximately 200 conserved segments (synteny blocks) between human and mouse and provided convincing arguments in favor of the random breakage model of genomic evolution postulated by Ohno in 1973 [2]. Further studies of significantly larger datasets (Copeland et al. in 1993 [3], DeBry and Seldin in 1996 [4], Burt et al. in 1999 [5], Lander et al. in 2001 [6], Mural et al. in 2002 [7]) with progressively increasing levels of resolution made the random breakage model the de facto theory of chromosome evolution and the Nadeau-Taylor predictions are viewed as among the most significant results in “the history and development of the mouse as a research tool” (Pennisi [8]).

The random breakage model implies that there are no evolutionary “faults” in mammalian genomes and rules out the systematic reuse of breakpoints from the same genomic regions. In 2003, Pevzner and Tesler [9] argued against the random breakage model by showing that any transformation of mouse gene order into human gene order would require a large number of breakpoint reuses. This conclusion immediately implies that the rearrangement breakpoints form clusters, a contradiction to the random breakage model. The Pevzner and Tesler arguments do not reveal the specific locations of the breakpoint regions where reuse occurred but instead give a nonconstructive combinatorial proof that these regions exist somewhere (at unknown locations) in the genome. Based on this result, Pevzner and Tesler rejected the random breakage model and proposed an alternative “fragile breakage” model of chromosome evolution. This model assumes the existence of fragile regions in genomes and postulates that the breakpoints occur mainly within these relatively short fragile regions (hot spots of rearrangements).

In 2004, Sankoff and Trinh [10] argued against the fragile breakage theory and raised doubts that Pevzner and Tesler [9] provided any proof of the breakpoint reuse phenomenon. They described an elegant computational experiment in which a series of random rearrangements creates the appearance of the breakpoint reuse phenomenon and argued that Pevzner and Tesler's arguments represent an artifact caused by microrearrangements and synteny block identification algorithms. Sankoff and Trinh [10,11] made an important contribution by raising awareness that synteny block determination is an important and nontrivial aspect of rearrangement analysis.

As a result, there is a controversy regarding the fragile breakage model. For example, Bailey et al. wrote in 2004 [12] that “our analysis supports a nonrandom model of chromosomal evolution that implicates a predominance of recurrent small-scale duplication and large-scale evolutionary rearrangements within specific fragile regions.” Similarly, Zhao et al. [13] wrote that “independent mathematical modeling of the syntenic block length distribution by us and others supports the fragile breakage model, but not the random breakage model for mammalian genome evolution.” On the other hand, Trinh et al. [14] wrote, “Indeed, there is no direct evidence for the fragile regions hypothesis, aside from the well-documented tendencies for rearrangements in pericentromeric and subtelomeric regions.”

The question then arises as to whether the Pevzner and Tesler [9] arguments were the first proof of breakpoint reuse or arguments without any merit. This question is ultimately connected to the question of whether Sankoff and Trinh [10] revealed errors in the Pevzner and Tesler arguments or whether the work by Sankoff and Trinh [10] represented an artifact. In this paper, we demonstrate that (1) the synteny block identification algorithm in [10] is flawed and (2) the parameters in [10] do not reflect the realities of the comparative genomic architecture of human and mouse. We show that if Sankoff and Trinh [10] fixed problems (1) and (2), their arguments against [9] would disappear.

In Section 1, we formally define the breakpoint reuse rate (BRR). In Section 2, we describe Sankoff and Trinh's synteny block identification algorithm, which we call ST-Synteny. In Section 3, we illustrate several shortcomings of this algorithm. In Section 4, we reproduce Sankoff and Trinh's simulations in which they applied ST-Synteny to synthetic genomes, and we perform the corresponding simulations using GRIMM-Synteny, obtaining different results for the effect of parameters on the breakpoint reuse rate. In Section 5, we show illustrations of the different blocks identified by ST-Synteny versus GRIMM-Synteny, using both synthetic data and using real human/mouse X chromosome anchors.

The simulation in Section 4 does not take into account issues from real-world data such as anchor lengths in nucleotides and variable length microrearrangements. In Section 6, we make an improved simulation that accounts for these issues and seed it with simulated genomes based on anchor lengths in the human/mouse X chromosomes and human/rat X chromosomes.

In Section 7, we give the results for whole genome comparisons of human and mouse, first based on alignments and then based on genes.

In Section 8, we analyze an intergenic breakage model, in which selection pressures prevent breaks occurring within genes and within regulatory regions upstream from genes.

## Results

### Section 1. Measuring the Breakpoint Reuse Rate

We study synteny blocks instead of conserved segments. Synteny blocks do not necessarily represent areas of continuous similarity between two genomes; instead, they may consist of many short regions of similarity interrupted by nonsimilar regions and gaps. Regions of similarity may be identified via homologous genes, anchors (alignments present in a single copy in both genomes), or other corresponding markers; we will call these *elements*.

When comparing genomes, rearrangements of the elements within a synteny block are called *microrearrangements,* or *microinversions* when we work in an inversions-only model. Rearrangements of the order of whole synteny blocks are called *macrorearrangements*.

Rearrangement distance is the minimum number of rearrangement operations required to transform one genome into the other; in the unichromosomal case, the operations are inversions, and in the multichromosomal case, the operations are inversions, translocations, fissions, and fusions.

Pevzner and Tesler's arguments [9] are based on computing the breakpoint reuse rate for the human and mouse genomes. The breakpoint reuse rate is computed as twice the rearrangement distance divided by the number of breakpoints, where these are computed as described in [15]. (We shall use the total number of breakpoints; a variant, using only internal breakpoints and excluding those at chromosome ends, was also used in [9] and [16] but will not be further considered here.)

The random breakage model implies a low breakpoint reuse rate (close to one, the minimum possible value for the breakpoint reuse rate), while the human/mouse rearrangement analysis revealed a very high breakpoint reuse rate (close to two, the maximum possible value for the breakpoint reuse rate). Based on this observation, Pevzner and Tesler rejected the random breakage model and proposed the fragile breakage model (which is consistent with a high breakpoint reuse rate) as a possible alternative.

### Section 2. ST-Synteny: Sankoff and Trinh's Synteny Block Identification Algorithm

Sankoff and Trinh's [10] simulations used a synteny block identification algorithm that we refer to as ST-Synteny. We emphasize that [10] used ST-Synteny to form synteny blocks, while [9] used a different algorithm, GRIMM-Synteny. While ST-Synteny appears to be a reasonable approach to synteny block identification, it was never applied to real data, only synthetic data. We compare ST-Synteny with GRIMM-Synteny on synthetic and real data.

GRIMM-Synteny is a parameter-dependent procedure that was designed to somewhat artificially separate microrearrangements and macrorearrangements found in real data. It was used for synteny block identification in the mouse [17], rat [16,18], and chicken [19,20] genomic projects. Since GRIMM-Synteny was first proposed in 2002, a number of other synteny block identification approaches were published and a recent study revealed that they largely agree [18]. Since GRIMM-Synteny was tested on many datasets and agreed with other approaches, we argue that it currently represents a consensus view on the synteny block identification approaches.

Below we show that ST-Synteny produces rather different results than GRIMM-Synteny. Since ST-Synteny produces results that are not consistent with the existing consensus views of comparative genomic architectures, we argue that their paper [10] revealed shortcomings of their own ST-Synteny algorithm rather than a flaw in [9].

Sankoff and Trinh's [10] arguments included a simulation of random rearrangements combined with their algorithm for synteny block identification (which we refer to as ST-Synteny). This process follows the random breakage model but somewhat surprisingly results in a high breakpoint reuse rate. They thus argued that the Pevzner-Tesler result has nothing to do with breakpoint reuse but is merely an artifact of the synteny block identification.

To compare ST-Synteny with GRIMM-Synteny, we first reproduced the procedure described in [10]. Although Sankoff and Trinh presented it as a single procedure, we break it into two phases: (1) a simulation to create synthetic rearranged genomes, which we give in Section 4, and (2) an algorithm to identify synteny blocks, which we present here.

Let π be a signed permutation on *n* elements (representing genes, anchors, or markers). This represents a genome that is rearranged as compared to a reference genome 1, … , *n*. Let *w,* Δ be positive integers; *w* represents the maximum microrearrangement span and Δ denotes the minimum number of elements in a synteny block.

#### ST-Synteny(π, w, Δ)

Step 1: Define each element of π as a block and iteratively amalgamate the resulting blocks as follows: two adjacent blocks in π are amalgamated if they contain elements *i* and *j,* where *i* or −*i* is in one block and *j* or −*j* is in another block and |*i* − *j*| ≤ *w*. Signs of the elements and blocks are recorded but ignored during amalgamation.

Step 2: Delete any “short” block containing less than Δ elements (Δ = 3 in [10]).

Step 3: Sankoff and Trinh [10] did not specify how signs of blocks were determined from their constituent elements. The block signs are necessary and important for calculating rearrangement distance. For example, reassignment of “random” signs (or assignment of all positive signs) to a permutation with low breakpoint reuse rate will lead (with high probability) to a permutation with high breakpoint reuse rate, thus emulating a breakpoint reuse artifact.

This implementation assigned signs to synteny blocks according to two different rules: the majority sign rule, where a block is assigned to the majority sign of all its elements; and the separable permutation rule described in [15]. The majority sign rule appears to be what [10] used (confirmed in private communication from Sankoff).

### Section 3. Shortcomings of ST-Synteny

We will show that ST-Synteny gives counterintuitive results even on small toy examples and then analyze the problems that occur as we scale up to larger synthetic or real-world datasets.

**Figure 1:**
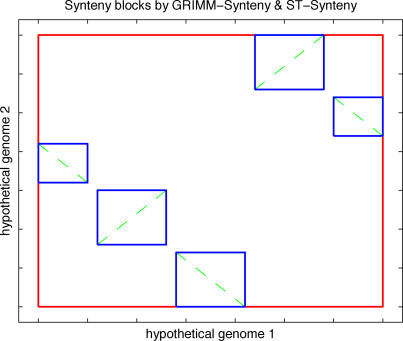
Nested Inversions Are Always Amalgamated by ST-Synteny The dot-plot of a signed permutation of anchors (green) between two genomes is shown. Since the anchors are signed, they are represented as ±45-degree segments. Blocks were constructed by ST-Synteny (red) and GRIMM-Synteny (blue). ST-Synteny amalgamates everything into one block. GRIMM-Synteny produces the correct blocks. If the genome on the horizontal axis is taken to be the identity permutation 1 2 3 4 5, then the genome on the vertical axis is the signed permutation −3 2 −1 −5 4.

The example in Figure 1 represents a genomic dot-plot for two fictitious genomes and illustrates one of the shortcomings of ST-Synteny. Finding synteny blocks for this permutation is a trivial problem and all other synteny block identification algorithms will output five synteny blocks, but ST-Synteny will form a single synteny block *for any setting of parameters*. Moreover, at the minimum setting of *w* = 1 (see below), ST-Synteny outputs a single block for 75% of all signed permutations of order 5! For other settings of the parameter *w,* the percentage of cases where ST-Synteny fails to reveal the correct synteny blocks increases even further.

We will describe in detail issues concerning of the granularity of blocks ST-Synteny forms. Given one inversion

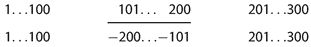



there should be three blocks and two breakpoints. However, there is no setting of the parameters of ST-Synteny that would do this. If *w* = 0, it is left as 300 separate elements (which are then deleted for being too small if Δ > 1). If *w* ≥ 1, ST-Synteny forms one large block, as follows. First, it performs a number of amalgamation steps resulting in the expected 3 blocks:

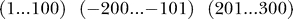
But the iterative amalgamation does not stop there. Since 100 and −101 are now in adjacent blocks and |101 − 100| = 1 ≤ *w,* it merges the first and middle blocks. Since −200 and 201 are in adjacent blocks, they are also merged. The result is a single block

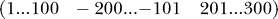
In fact, a signed permutation produced by applying any series of nested inversions to the identity will be reamalgamated into a single block by ST-Synteny whenever *w* ≥ 1. (For nonnested inversions, reamalgamations may also occur, resulting in fewer blocks than appropriate. For *w* = 1 and large *n,* this happens in ST-Synteny for over 77% of the signed permutations of length *n* that would not have any reamalgamation if the signs were properly considered. A paper is in preparation.) Inversions are *nested* when the two breakpoints of each inversion both lie within any single existing strip (as defined by the previous inversions) *i, i* + 1, *i* + 2, … , *j* or −*j,*−(*j* − 1), … , −*i*; “lie within” includes reuse of either or both endpoints of the strip. For example, consider this series of inversions:

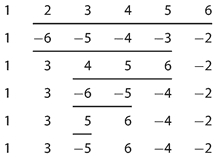



When given the last line as input, ST-Synteny first combines −5 and 6 into a block (−5 6). That block is amalgamated with the −4 into (−5 6 −4). Then 3, −2, 1 are successively amalgamated into that, resulting in a single block (1 3 −5 6 −4 −2).

In addition to the above-mentioned problems, if microrearrangements have occurred or if there is noise in the form of spurious elements, ST-Synteny tends to compute several smaller blocks rather than a larger block with microrearrangements. ST-Synteny assumes that all markers (orthologous genes or sequence anchors) are direct descendants of the most recent common ancestor of the two organisms being compared. This may be a reasonable assumption in comparing mitochondrial, chloroplast, or viral genomes, but between free-living organisms, false orthologs are inevitable, no matter how carefully the annotations are curated. Since the computed blocks are smaller, it increases the chance of them being deleted for being too short. It nonetheless increases the number of reported blocks, decreases their size, and increases the length of the breakpoint regions as compared to GRIMM-Synteny, thus tending to increase the number of breakpoint reuses even for “random” rearrangements. One may argue that this excessive granularity can be fixed by simply changing the parameters of ST-Synteny, but as we showed above there is often no choice of parameters that resolves the problem.

The reason is spurious elements (which are shown as black dots in the X chromosome illustration later in this paper in Section 5). If ST-Synteny is given a permutation with stray points 200 and 300

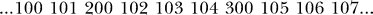
then (at small *w* and Δ = 3) it first forms blocks


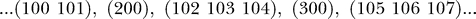


Then it deletes small blocks (100 101), (200), (300), leaving two blocks: (102 103 104) and (105 106 107). (There is no rearrangement breakpoint between these two blocks, however.)

By contrast, GRIMM-Synteny does not require the elements in a block to be contiguous in all species in the input data. It first forms blocks

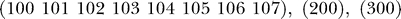
and then it deletes the small blocks (200) and (300), leaving one large block.

**Figure 2:**
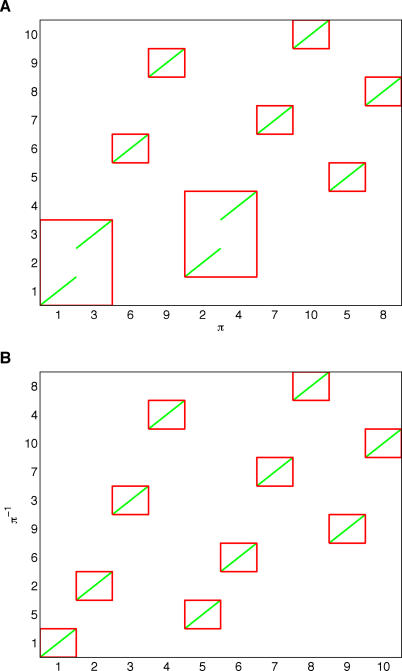
Asymmetric Treatment of Genomes by ST-Synteny In comparing two genomes, ST-Synteny may produce different synteny blocks depending on which one is chosen as the reference genome. The synteny blocks produced by ST-Synteny are shown as red boxes around the anchors. (A) The genome shown on the *y*-axis is the reference genome 1, …, 10, and the genome shown on the *x*-axis is represented as a permutation π of this. (B) The exact same anchor arrangement is shown, but the *x*-axis is taken as the reference genome 1, …, 10 and the *y*-axis is the permutation π^−1^. Although the anchor arrangements are identical, ST-Synteny with parameters *w* = 2, Δ = 1 produces different blocks depending on which genome is the reference genome.

Another problem is that a synteny block identification algorithm should produce the same blocks when the two genomes are swapped. ST-Synteny, however, is not always symmetric in the two genomes when *w* ≥ 2. For example, consider the following permutation and its inverse permutation:

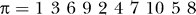


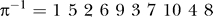
At *w* = 2, the blocks output by ST-Synteny for π are

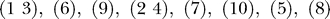
while the blocks output for π^−1^ are

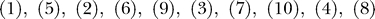
This is illustrated in Figure 2.

### Section 4. GRIMM-Synteny versus ST-Synteny

In the previous sections, we described ST-Synteny, the synteny block identification algorithm within Sankoff and Trinh's simulations. They generated synthetic genomes to input to it as follows:

#### Simulation *(n, m, k, w)*

Step 1: Generate m = 150 random inversions of a reference genome 1, 2, … , *n* with *n* = 5,000 “genes” (elements).

Step 2: Generate *k* microinversions of exactly *w* elements, each randomly placed throughout the genome.

Step 3: Output the resulting signed permutation π of *n* elements.

Then they apply ST-Synteny(π, w, Δ) to identify synteny blocks. Finally, they apply the GRIMM algorithm [21,22] to the resulting synteny blocks to calculate the rearrangement distance and the breakpoint reuse rate.

We reproduced the Sankoff-Trinh random simulations with GRIMM-Synteny in place of ST-Synteny to verify whether the appearance of the breakpoint reuses persists under the random breakage model. To compare ST-Synteny with GRIMM-Synteny on the same simulated permutations, we had to determine the parameters that best match the parameters used in Sankoff and Trinh [10]. This is a nontrivial task since ST-Synteny is quite different from all other synteny block identification algorithms. We also remark that the description of ST-Synteny lacks some important details. For example, while Pevzner and Tesler [15] emphasized the importance of signs in the synteny block analysis, Sankoff and Trinh [10] do not even address this important issue.

GRIMM-Synteny(π, *G, C*) has maximum gap size parameter *G* (which is similar in spirit to *w* in ST-Synteny) and minimum cluster span parameter *C* (which is similar in spirit to Δ in ST-Synteny). For comparison, ST-Synteny(π, w, Δ) was replaced by GRIMM-Synteny(π, G, C) with “similar parameters,” to identify synteny blocks and compute reversal distance and breakpoint reuse rates. While we tried our best to match ST-Synteny and GRIMM-Synteny parameters, we emphasize the following concerns.

Our first concern is that Sankoff and Trinh regard elements (genes or anchors) as individual points with length 1, and while the element orientations are recorded, they do not make use of the orientations. Under these circumstances, a gap threshold *G* = *w* + 3 and minimum block size *C* = Δ is the best match.

**Figure 3:**
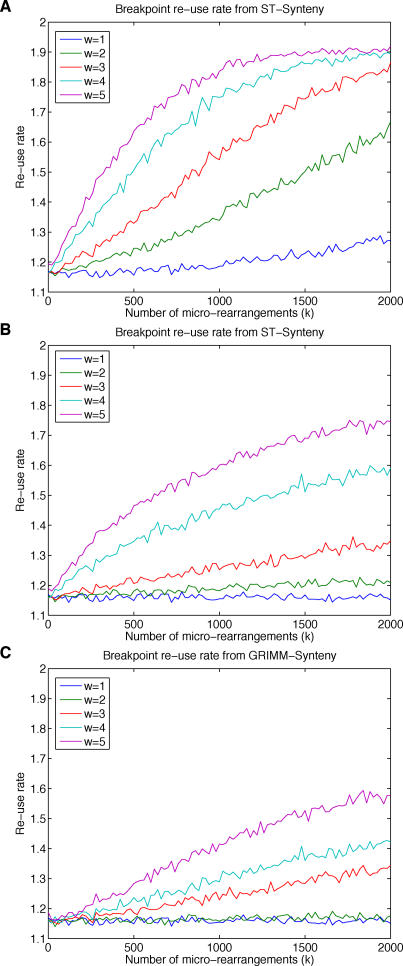
Breakpoint Reuse Rates in Simulations The simulated number of microrearrangements is *k,* and the microrearrangement size is *w.* The same simulated rearrangements were analyzed three ways. (A) ST-Synteny simulation, with signs of blocks determined using their majority sign rule. (B) ST-Synteny simulation, with signs of blocks determined using GRIMM-Synteny's separable permutation rule. (C) GRIMM-Synteny simulation. Anchors have length 1 for comparison with ST-Synteny.

In our simulations in Figures 3, S1, and S2, we used a minimum of Δ = 3 anchors per block in both programs, instead of GRIMM-Synteny's original definition of *C* as the minimum size in terms of the “span” of a block; the plots (not shown) on using the original definition of *C* = 3 in GRIMM-Synteny are similar to those shown using Δ = 3 instead. This isolates the differences in performance of the algorithms to the methods used to join elements into blocks.

Our second concern is that ST-Synteny does not make use of element signs or lengths, or gaps between elements; GRIMM-Synteny does take them into account, and it does affect whether the elements should be amalgamated into one block. Normally, when dealing with gene orders with unknown gene lengths and unknown intergenic gap lengths, GRIMM-Synteny would assign a length of 2 to each gene (element) so as to make the two ends distinguishable. However, the simulations in Figures 3, S1, and S2 are done with length 1 anchors in both programs for a more direct comparison.

Breakpoint reuse rate as a function of the number of microinversions is shown in Figure 3A (ST-Synteny, majority sign rule), 3B (ST-Synteny, separable permutation sign rule), and 3C (GRIMM-Synteny). Note that at *k* = 0, there is no microinversion. Although a microinversion of span *w* = 1 changes the sign of an element, it is ignored by ST-Synteny, so that *w* = 1 serves only as the threshold for block amalgamation.

Notice that as the number of microinversion increases and the span of inversion increases, the breakpoint reuse rate of simulation with ST-Synteny increases much faster than that of GRIMM-Synteny. Sankoff and Trinh [10] simulated microinversion spans of up to ten or 15 elements, which correspond to approximately 6 and 9 Mb of the human genome, respectively; this is much larger than the average inversion span of microrearrangements between human and mouse computed by GRIMM-Synteny. These unrealistically large spans are therefore excluded from our simulations. To put things in perspective, the average size of microrearrangements in the human/mouse comparison is 196 kb, corresponding to “*w* = 0.33” (although *w* can only be an integer), and the median microrearrangement size is approximately 7 kb. It implies that the most realistic simulations in Sankoff and Trinh [10] correspond to very small *w* (although even *w* = 1, 2 correspond to rearrangements with longer average span than in the case of human/mouse comparison). For these parameters and with a correct synteny block identification algorithm, the breakpoint reuse rate is low for the random breakage model, negating the Sankoff-Trinh arguments against the fragile breakage model.

To further contrast the two models, the percentages of the breakpoint regions of the whole simulated genome, denoted as *bk,* were computed and are shown in Figure S1 for ST-Synteny and GRIMM-Synteny. The breakpoint regions from ST-Synteny are much larger as compared to GRIMM-Synteny, thus refuting another Sankoff-Trinh argument against the random breakage reuse phenomenon (the larger the breakpoint regions, the better are the chances to observe breakpoint reuse, even in the random breakage model).

The number of elements kept by each model is shown in Figure S2. As the number of microrearrangement grows, ST-Synteny deletes elements at a faster rate than GRIMM-Synteny, as a result of deleting small blocks. That ST-Synteny outputs fewer blocks than GRIMM-Synteny for relatively small (although still unrealistically large from the perspective of real human/mouse genomic architectures) *w* and large *k* (unpublished data) can be explained by the combination of deleting small blocks and mistakenly amalgamating large ones.

### Section 5. GRIMM-Synteny versus ST-Synteny: Dot-Plots

**Figure 4:**
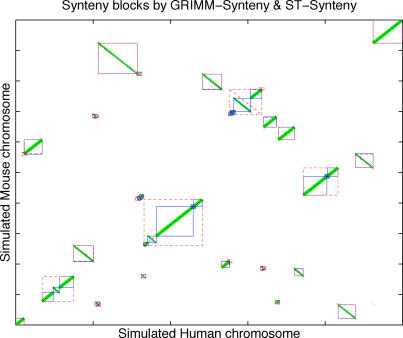
GRIMM-Synteny and ST-Synteny on the Same Simulated Data The genomic dot-plot is shown in thick green. The synteny blocks identified by GRIMM-Synteny are shown as blue rectangles, and the ones from ST-Synteny are dashed red rectangles. When block coordinates coincide, this appears as dashed blue/red. Signs of the blocks are shown as diagonals. Tiny blocks have been artificially enlarged for visibility and do not actually protrude into other blocks. The simulated human genome has anchors 1 through 5,000. The simulated mouse genome was generated as π = Simulation(5000, 15, 500, 5). Blocks were identified via GRIMM-Synteny(π, 8, 3) and ST-Synteny(π, 5, 3).

**Figure 5:**
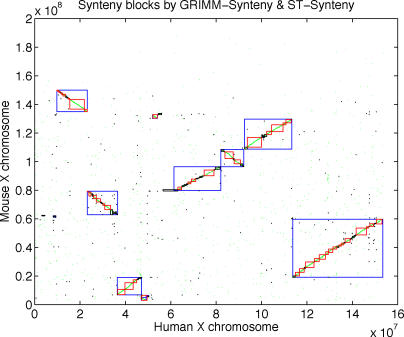
Synteny Blocks between Human and Mouse X Chromosomes Blocks for the X chromosomes were constructed by GRIMM-Synteny (blue) based on anchor coordinates and ST-Synteny (red) based only on anchor permutations. Anchors are shown in green. Small blocks deleted by ST-Synteny are shown in black.

We illustrate some of the differences between ST-Synteny and GRIMM-Synteny with a synthetic dataset (Figure 4) and real human/mouse data (Figure 5).

We constructed a synthetic genome π = Simulation(5000, 15, 500, 5). The genomic dot-plot and synteny blocks output by ST-Synteny and GRIMM-Synteny for this genome with one setting of detection parameters are shown in Figure 4. The breakpoint reuse rate is 1.31 and 1.09 from blocks output by ST-Synteny and GRIMM-Synteny, respectively. Notice that ST-Synteny amalgamated blocks that should be separate. For each *w* from 1 to 5, we made ten simulated genomes π = Simulation(5000, 15, 500, *w*). The average breakpoint reuse rate from ST-Synteny(π, *w,* 3) rises from 1.07 to 1.30, while in GRIMM-Synteny(π, *w* + 3, 3), the breakpoint reuse rate increases only from 1.03 to 1.09.

To further illustrate the difference between ST-Synteny and GRIMM-Synteny in identifying synteny blocks, both methods were applied to human/mouse anchors on the X chromosome (National Center for Biotechnology Information [NCBI] Human version 34, Mouse version 30). There are 58,930 anchors, and the human X chromosome has a length of 153,692,391 base pairs (bp). The full version of GRIMM-Synteny, which accounts for anchor coordinates, lengths, and signs, was used with minimum block size threshold of 1 Mb and maximum gap threshold of 1 Mb. ST-Synteny was run with *w* = 378 and Δ = 379. Since ST-Synteny uses only the permutation of the anchors, we plot the resulting blocks from both programs together superimposed with anchor permutations. Results are shown in Figure 5 and Table 1. GRIMM-Synteny identified ten blocks with microinversion distance of 825. ST-Synteny identified many more synteny blocks than did GRIMM-Synteny. The number of blocks in ST-Synteny did not reduce dramatically when the threshold *w* was increased (data not shown).

**Table 1:**
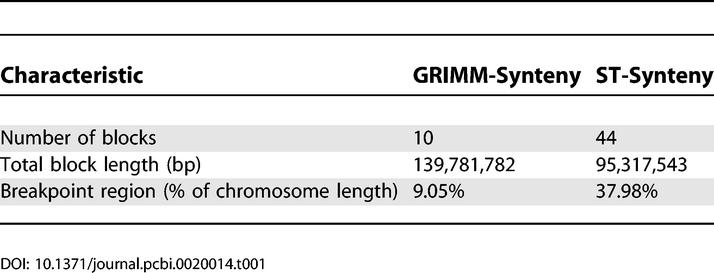
GRIMM-Synteny versus ST-Synteny Applied to Human and Mouse X Chromosomes

If one compares Figures 4 and 5, one will notice that ST-Synteny outputs more blocks than GRIMM-Synteny in Figure 5 but fewer in Figure 4. Neither the order of magnitude difference in the number of markers (5,000 genes versus 59,000 anchors) nor the random versus fragile breakage model is responsible for this “role reversal.” As noted in Section 3, one of the tendencies of ST-Synteny is to amalgamate blocks that should be separate, and another is its inability to merge synteny blocks in the presence of short “out-of-place” inversions or false orthologs. In Figure 4, the numbers of macrorearrangements and microrearrangements were chosen to be much smaller than the previous simulations in order for the synteny blocks to be legible; in this case, the first deficiency of ST-Synteny dominates, resulting in fewer blocks than that from GRIMM-Synteny. When the number of rearrangements becomes large or when data become noisy (as in the case of real genomic data [Figure 5]), the second deficiency of ST-Synteny dominates. By contrast, GRIMM-Synteny was designed to filter out noise, thereby identifying more realistic synteny blocks.

### Section 6. Improved Simulation including Varying Length Anchors and Microrearrangements

In the previous simulations, the length and coordinate distributions of anchors were ignored, and permutations were performed on unit-length anchors (elements). To better simulate randomized rearrangements, GRIMM-Synteny was applied to simulated unichromosomal and multichromosomal genomes with anchors of varying lengths. In the simulated genome, randomized scenarios of rearrangements were generated at the level of nucleotides. The details of the simulation are as follows.

Step 1: Take the human coordinates of human/mouse alignment anchors derived from NCBI Human version 34 and Mouse version 30. In the unichromosomal genome simulation, anchors from the X chromosome were used. In the multichromosomal genome simulation, all anchors were used.

Step 2: For the unichromosomal simulation, generate six inversions at random locations. Inversions in this genome represent true inversions since “What breaks in the X chromosome, stays in the X chromosome.” For the multichromosomal simulation, generate 150 pairs of breakpoints at random locations in the genome. When a pair of breakpoints lie on the same chromosome, perform an inversion, and when they lie on different chromosomes, perform a translocation. If a breakpoint resides within an anchor, the breakpoint is moved at random to the immediate left or right of the anchor; anchors are not split.

Step 3: Generate *k* microinversions randomly located in the genome. The spans of inversions are randomly distributed between 1 and *W* nucleotides. Since the distribution of the spans of inversions is unknown, the uniform distribution used in the simulation is chosen somewhat arbitrarily.

Step 4: Add noise to the simulated genomes as follows. Randomly choose 0.2% of the anchors to be noisy anchors. Move each noisy anchor to a random location in the genome and chose its sign at random.

Step 5: Apply the GRIMM-Synteny algorithm to identify synteny blocks and compute the inversion or multichromosomal distance and breakpoint reuse rate between the human and the simulated genomes. Both block size and gap thresholds were set to 1 Mb.

The results of the unichromosomal simulation are listed in Table 2, and the results of the multichromosomal simulation are listed in Table 3. As in the previous section, *bk* is the percentage of the genome in breakpoint regions. *r_e_* is the percentage of blocks that have at least one of the two ending anchors of the simulated genome out of order as compared to those of the original human anchors of the block.

**Table 2:**
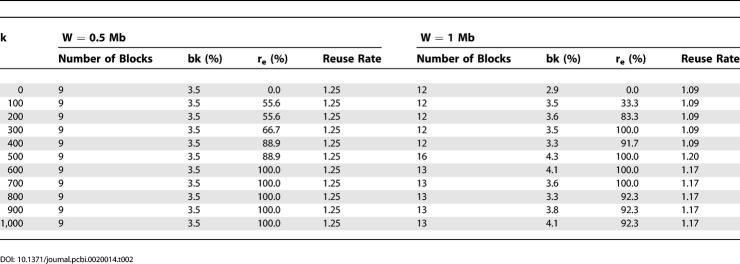
Randomized Unichromosomal Rearrangement Simulation with Human X Chromosome Anchors

**Table 3:**
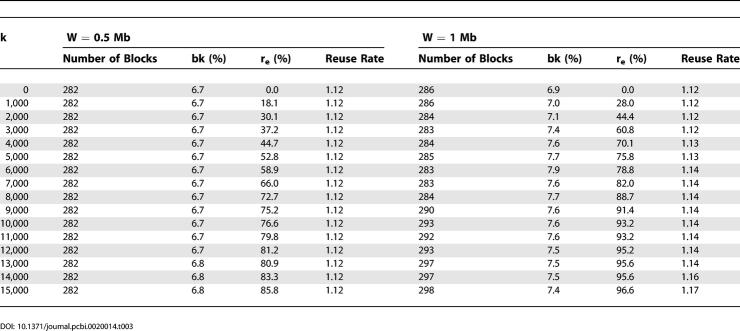
Randomized Multichromosomal Rearrangement Simulation with Human Anchors

A similar simulation was also performed on mouse/rat X chromosome alignment anchors, which presumably have more signal and less noise than human/mouse alignment anchors. The anchors were derived from mouse NCBIM33 and rat RGSC3.4 using BLASTZ [23]. GRIMM-Synteny identified 18 synteny blocks, with microinversion distance of 4,287. The breakpoint reuse rate is 1.58. Using these parameters as a reference, the simulated genomes started with the mouse coordinates of the mouse/rat X chromosome anchors, followed by nine macroinversions and up to 5,000 microinversions based on the random breakage model. GRIMM-Synteny was applied to the resulting genomes. The results are listed in Table 4. Notice that when the maximum inversion span is *W* = 1 Mb, the breakpoint reuse rate is mostly 1.00, the theoretical value under the random breakage model.

**Table 4:**
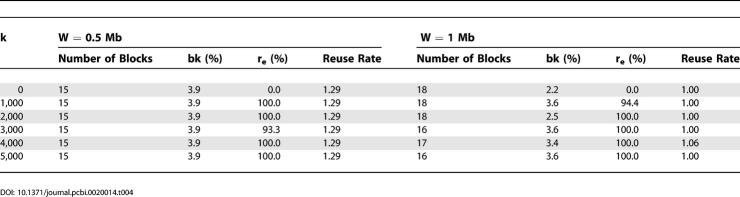
Randomized Unichromosomal Rearrangement Simulation with Mouse X Chromosome Anchors from Mouse/Rat Alignment

One may notice that in Table 3, the reuse rate is at least 1.12 under random breakage model while theoretically it should be 1.00. The difference is explained by the block size and gap thresholds used in GRIMM-Synteny. To show the effect of thresholds on the breakpoint reuse rate, GRIMM-Synteny was performed on the simulated dataset with 150 macroinversions and without microinversion. The block size and gap threshold were decreased from 1 Mb to 10 kb. The resulting breakpoint reuse rates are shown in Table 5. As the threshold decreases from 1 Mb to 10 kb, the breakpoint reuse rate decreases from 1.12 to 1.02. The side effect of the thresholds is small, however, and does not itself explain the high breakpoint reuse rate found in real human/mouse genome data if the random breakage model is to be assumed.

**Table 5:**
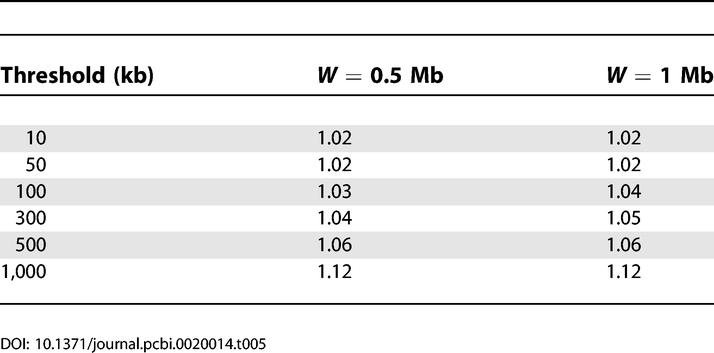
Breakpoint Reuse Rate versus Block Size and Gap Threshold Used in GRIMM-Synteny on Simulated Genome-Based Random Breakage Model

### Section 7. Analyzing Whole-Genome Rearrangements in Human and Mouse

All of the simulations, whether permutation or sequence based, were based on randomized rearrangements. How do they compare to human/mouse rearrangements? Applying the GRIMM-Synteny algorithm to the real human/mouse alignment data with block size and gap threshold of 1 Mb yielded 294 synteny blocks and genome distance of 262. The breakpoint reuse rate was 1.67.

We further studied the starting and ending anchors within each synteny block to arrive at realistic parameters for microrearrangements. In the absence of microrearrangements, the ending anchors of every synteny block are conserved between two genomes. Microrearrangements acting on ends of synteny blocks destroy this conservation; e.g., an ending anchor in a human synteny block may not be an ending anchor in a mouse synteny block. The percentages of blocks that have at least one of the two ending anchors out of order is denoted as *r_e_*. Of 294 human/mouse synteny blocks, 115 had anchors at one or both ends out of order; together these give an *r_e_* of 39.1%.

The 294 human/mouse synteny blocks contained 10,900 microrearrangements. Of these microrearrangements, the average span of inversion was 196 kb, the median was 7 kb, and the maximum was 13.9 Mb. In terms of the number of anchors, the average span of inversion was 78, the median was four, and the maximum was 7,632. The breakpoint regions comprised 9.06% of the human genome. For a comparable breakpoint reuse rate, the span of microrearrangements would have to be unrealistically large. The values of *k* and *r_e_* in the human/mouse data are much higher and the value of *bk* is much lower than those from the simulated genomes generated with the random breakage model. This may suggest that rearrangements, or breakpoints, are not randomly distributed across the whole genome.

As shown in the randomized rearrangement simulations, breakpoint reuse increased with the number of microrearrangements and the length of microinversions. In sequence-based human/mouse rearrangement analysis, approximately 10,000 microrearrangements were found inside 294 synteny blocks. When the human/mouse comparison was performed based on gene order, only 98 microrearrangements were found inside 373 synteny blocks. The breakpoint reuse rate of microrearrangements was only 1.02. Data from sequence- and gene order–based comparison are listed in Table 6.

**Table 6:**
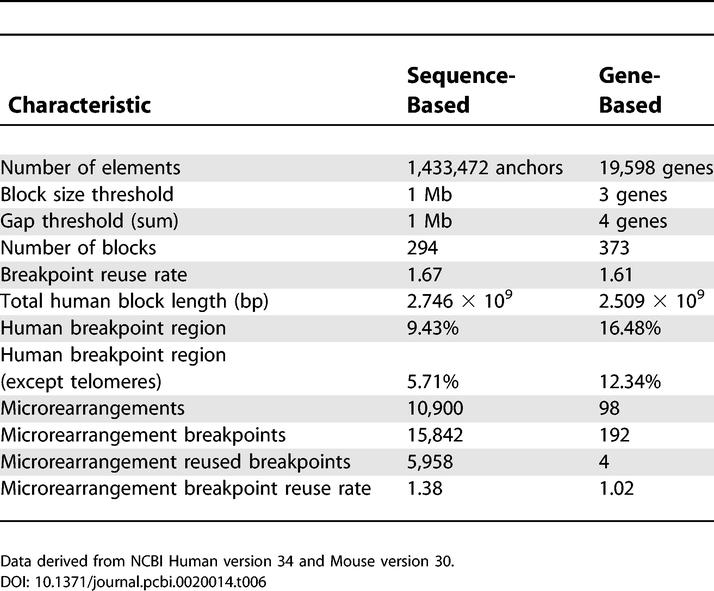
Sequence-Based versus Gene-Based Rearrangements

### Section 8. Intergenic Breakage Model

**Figure 6:**
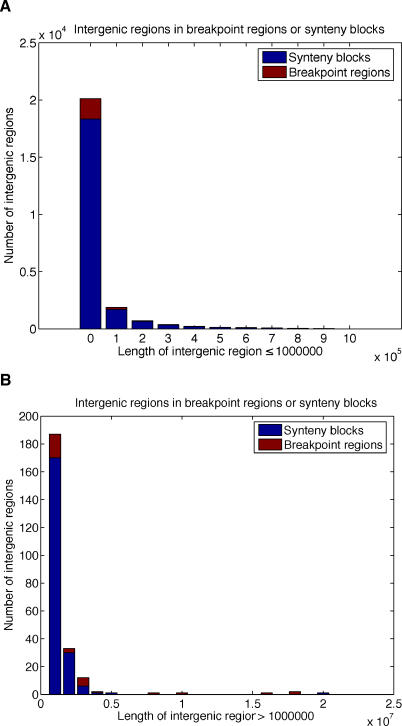
Distribution of Human Intergenic Regions within Synteny Blocks or within Breakpoint Regions (A) Regions of length ≤1 Mb and (B) length >1 Mb that are within synteny blocks (blue) and within breakpoint regions or across breakpoint regions and synteny blocks (red). Data derived from NCBI Human version 34 and Mouse version 30.

What makes certain regions break and others not? Is it a biophysical constraint or a selection constraint? One observation we can make is that breaks are less likely to occur within genes (and within regulatory regions) since selection usually works against such breaks. The average length of intergenic regions of the human genome (NCBI version 34) is approximately 80 kb, much smaller than the average length of breakpoint regions. There are a total of 21,911 intergenic regions within synteny blocks, with an average length of 77 kb, while the average length of the 2,116 intergenic regions within breakpoint regions is a much higher 100 kb. The distributions of length of human intergenic regions are shown in Figure 6. While the majority of the intergenic regions are short and reside within synteny blocks (Figure 6A), notice in Figure 6B that of 241 long intergenic regions with length greater than 1 Mb, 32 reside within breakpoint regions or across breakpoint regions and synteny blocks. If we assume that breakpoints occur randomly within intergenic regions (and are nearly forbidden inside the genes), then the gene-dense regions with small overall lengths of intergenic regions will rarely break simply by chance, thus mimicking what was referred to as “solid regions” in [9].

To investigate the hypothesis that long intergenic regions may be potential hot spots of rearrangements, we performed the following simulation. Assume that the genome consists of *G* + *I* nucleotides, where *G* is the total size of genes extended by upstream regulatory regions and *I* is the total size of remaining intergenic regions. To account for upstream regulatory regions (which are also unlikely to be broken), we artificially extended every gene by a region of length *R* upstream, thus reducing the sizes of corresponding intergenic regions by *R* nucleotides. Although regulatory regions in mammalian genomes are often located far from the gene starts, the average size of such regulatory regions remains unknown. We remark that genome rearrangement studies may shed light on the size of regulatory regions. For example, if there was a rearrangement (or even a microrearrangement) between human and chimpanzee *R* nucleotides from a gene start, it is likely that the regulatory region for this gene is shorter than *R* (otherwise, the rearrangement would disrupt the regulatory region). Although such conclusions should be taken with caution (e.g., they do not apply if the human and chimpanzee genes exhibit very different regulatory patterns), they are useful as the first approximation for the otherwise difficult problem of delineating regulatory regions. Below we address an even more difficult problem of estimating the average size of regulatory regions *R* based on rearrangement analysis.

**Figure 7:**
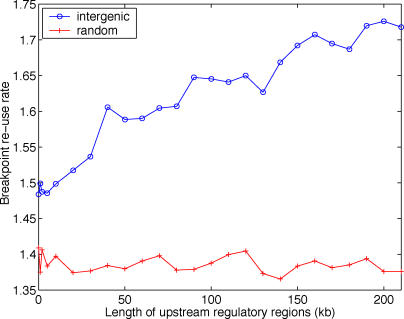
Breakpoint Reuse Rates as a Function of Upstream Regulatory Region Size in Intergenic Breakage Model Simulations Genes from NCBI Human version 34 were each extended by length 0 to 210 kb upstream, thus shortening or eliminating the intergenic regions. In the intergenic breakage model, simulated reversals were performed with breakpoints chosen uniformly among the nucleotides remaining in the shortened intergenic regions, while in the random breakage model, breakpoints were chosen uniformly among all nucleotides in the genome. Then blocks were derived, and the breakpoint reuse rate was computed.

We remark that if the length of regulatory regions is set to be *R,* the intergenic regions shorter than *R* may disappear (depending on the orientation of the genes), thus leading to “merging” genes separated by short intergenic regions and promoting breakpoint reuse elsewhere. As a first approximation, we assume that the probability of breakage at each nucleotide within genes and regulatory regions is 0 and the probability of breakage at each nucleotide in intergenic regions is 1/*I* (note that *I* decreases as *R* increases). We performed 240 random macrorearrangements with breakpoints chosen by this distribution and applied GRIMM-Synteny (using minimum block size threshold of 1 Mb and maximum gap threshold of 1 Mb) to compute blocks and then the breakpoint reuse rate. We contrasted this *intergenic breakage model* against the standard random breakage model (same simulation except the probability of breakage is uniform at all nucleotides in the genome). Figure 7 shows how the breakpoint reuse rate changes as the size, *R,* of regulatory regions is increased. One can observe that breakpoint reuse rate is already significant (approximately 1.5) even for relatively modest sizes of regulatory regions. Moreover, for *R* ≈ 90 to 140 kb, the breakpoint reuse rate is similar to the human/mouse breakpoint reuse rate of 1.65 we reported in [16]. We therefore argue that long regulatory regions and inhomogeneity of gene distribution in mammalian genomes may provide at least a partial explanation for the fragile breakage model. A more realistic explanation, however (in view of the fact that the estimate *R* ≈ 90 to 140 kb seems to be rather high), is that a combination of long regulatory regions, uneven distribution of sizes of intergenic regions, and other (still unknown) factors triggers high breakpoint reuse.

## Discussion

The GRIMM-Synteny algorithm in Pevzner and Tesler [9] is a parameter-dependent procedure that was designed to somewhat artificially separate microrearrangements and macrorearrangements. An important contribution of Sankoff and Trinh [10] is to draw attention to the fact that these parameters may affect the robustness of the rearrangement analysis. There is no doubt that discarding small blocks affects the rearrangement inference and offsets the breakpoint reuse calculations. This paper studies the question of whether this offset is large enough to create an appearance of a large breakpoint reuse even in the random breakage model. We demonstrate that this offset is relatively small as compared to the Sankoff and Trinh [10] computational experiment and explain why our simulation and the Sankoff and Trinh [10] simulation disagree.

The Pevzner and Tesler [9] work is based on the reversals/translocations/fusions/fissions model of genome rearrangements and ignores other types of rearrangements, e.g., large-scale transpositions, which are believed to be rare. They do not attribute the breakpoint reuse phenomenon to a particular type of rearrangement and do not rule out the possibility that a significant portion of breakpoint reuse is caused by yet another rearrangement operation. For example, if one assumes that transpositions are frequent, then they may account for a significant portion of breakpoint reuse since every transposition creates three (rather than two) breakpoints, immediately increasing breakpoint reuse rate as we defined it. In this case, an analysis of giant cycles in the breakpoint graph may be necessary to evaluate the breakpoint reuse rate parameter (a series of transpositions in a random model would not lead to the giant cycles that we observed in the human/mouse breakpoint graph).

Evolutionary breakpoints are often confused with cancer breakpoints. We emphasize that the well-established recurrent breakpoints in cancer and infertility have nothing to do with evolutionary breakpoints and do not provide corroborative evidence for fragility in an evolutionary context. Pevzner and Tesler [9] did not answer the question, “Where are the recurrent breakpoints in mammalian genomes?” and studies are under way to determine comparative genomics and phylogenetic evidence for breakpoint reuses. For example, Murphy et al. [24] recently analyzed genomic architectures for eight mammalian genomes derived from either sequence or large-scale radiation hybrid mapping experiments and observed breakpoint reuse in independent lineages.

Another open question is how many fragile sites are in the human genome. Pevzner and Tesler [9] did not rule out the possibility that most breakpoint reuses are caused by very few fragile sites. For example, one can envision a model with just one or two fragile sites with all rearrangements having one end in the fragile region and another end chosen randomly. Such a fragile hubs model is not inconsistent with the Pevzner and Tesler [9] analysis. Studies of giant cycles in the breakpoint graph may again shed light on whether the fragile hubs model is correct.

Sankoff and Trinh's work is an insightful contribution to studies of chromosome evolution that raised awareness about the importance of synteny block identification and microrearrangement analysis. Unfortunately, their rebuttal of the breakpoint reuse phenomenon was plagued by shortcomings of their own synteny block identification algorithm and unrealistic choices of parameters in their simulation procedure. We therefore argue that the breakpoint reuse phenomenon is real until the next critical argument against it emerges.

## Materials and Methods

We repeated Sankoff and Trinh's simulation [10] by breaking it into two parts, Simulation and ST-Synteny, as described in Section 2 of this paper.

We compared the synteny block identification algorithms of Pevzner and Tesler's with those of Sankoff and Trinh on simulated data from Simulation (Section 4) using GRIMM-Synteny [9] and ST-Synteny (Section 2). Breakpoint reuse rates were computed with GRIMM [21,22].

In the analysis and simulations based on real genome data (Section 7), we used human/mouse anchors derived from NCBI Human version 34 and NCBI Mouse version 30. Mouse/rat X chromosome anchors were derived from NCBI Mouse version 33 and the Rat Genome Sequencing Consortium assembly RGSC version 3.4. NCBI Human version 34 was also used in the intergenic breakage model simulation (Section 8).

## Supporting Information

Figure S1 Breakpoint Region Size in Simulations Ratio of length of breakpoint region to length of genome in simulations as a function of number of microrearrangements *k* and microrearrangement size *w* for (A) ST-Synteny and (B) GRIMM-Synteny.
(11 KB PDF)

Figure S2 Number of Elements Retained in Simulations after Deletion of Small Blocks Number of elements retained by (A) ST-Synteny and (B) GRIMM-Synteny, as a function of the number of microrearrangements *k* and microrearrangement size *w* in the simulated data.
(12 KB PDF)

## Abbreviations

bp: base pair
NCBI: National Center for Biotechnology Information
