# Supplementary material for: The Fragile Breakage versus Random Breakage Models of Chromosome Evolution
Source: PLoS Comput Biol. 2006 Feb 24;2(2):e14. doi: 10.1371/journal.pcbi.0020014 (PMC1378107; doi:10.1371/journal.pcbi.0020014)
Supplement: Supplementary file 1 [file pcbi.0020014.sg001.pdf]

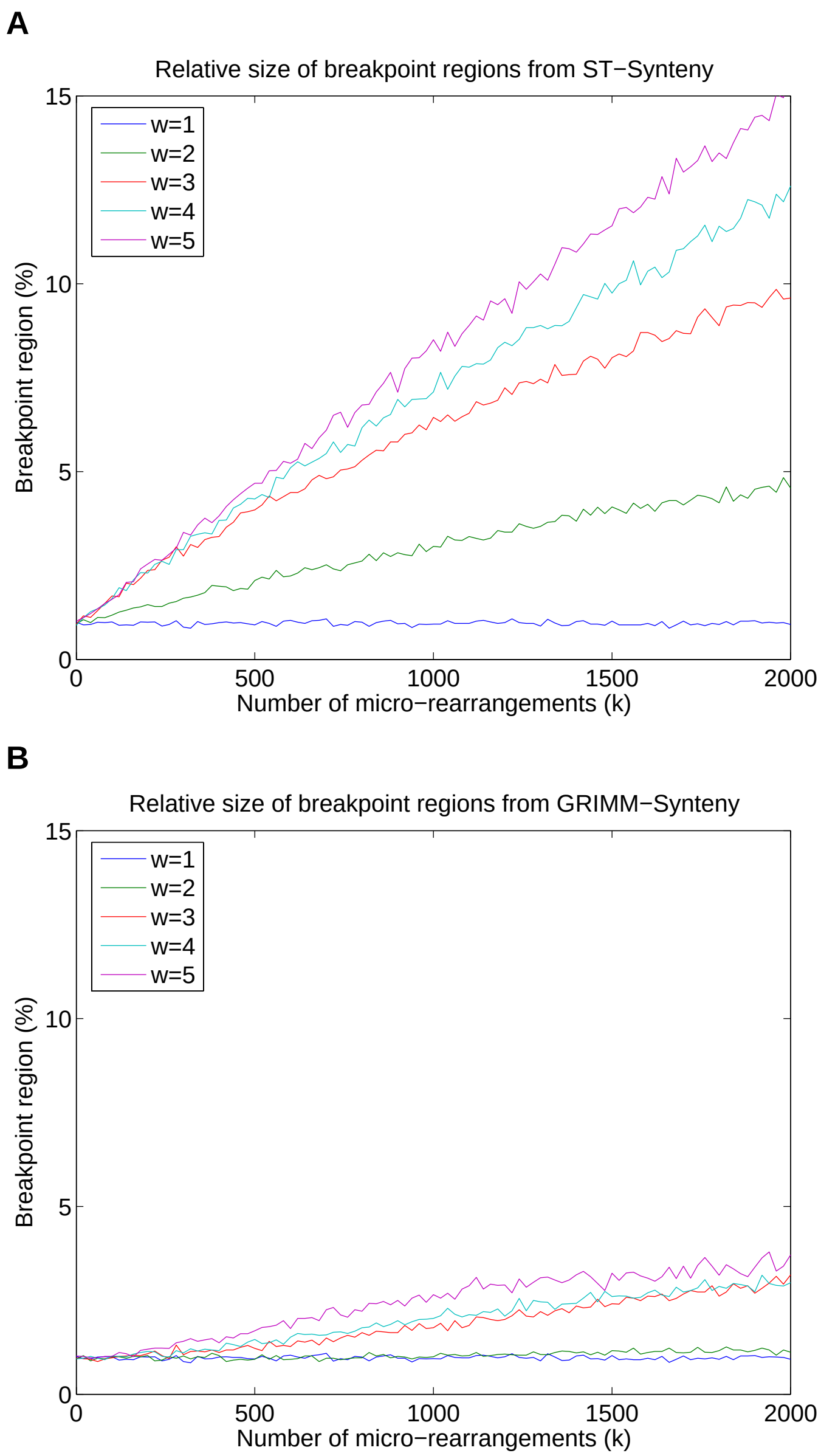

Figure S1. Breakpoint region size in simulations. Ratio of length of breakpoint region to length of genome in simulations as a function of number of micro-rearrangements k and micro-rearrangement size w, for (A) ST-Synteny, and (B) GRIMM-Synteny.
