# Supplementary material for: The Fragile Breakage versus Random Breakage Models of Chromosome Evolution
Source: PLoS Comput Biol. 2006 Feb 24;2(2):e14. doi: 10.1371/journal.pcbi.0020014 (PMC1378107; doi:10.1371/journal.pcbi.0020014)
Supplement: Supplementary file 2 [file pcbi.0020014.sg002.pdf]

A

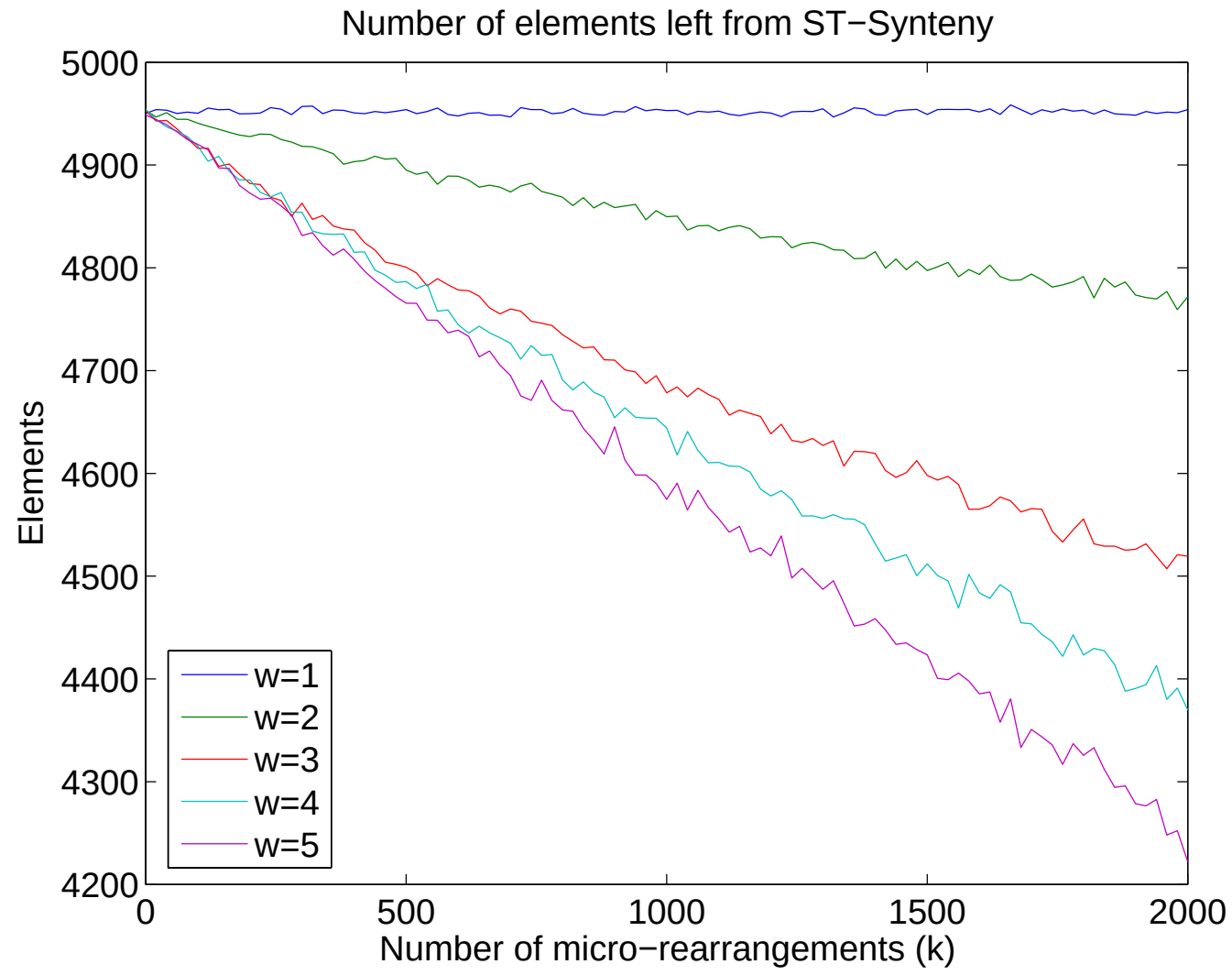

B

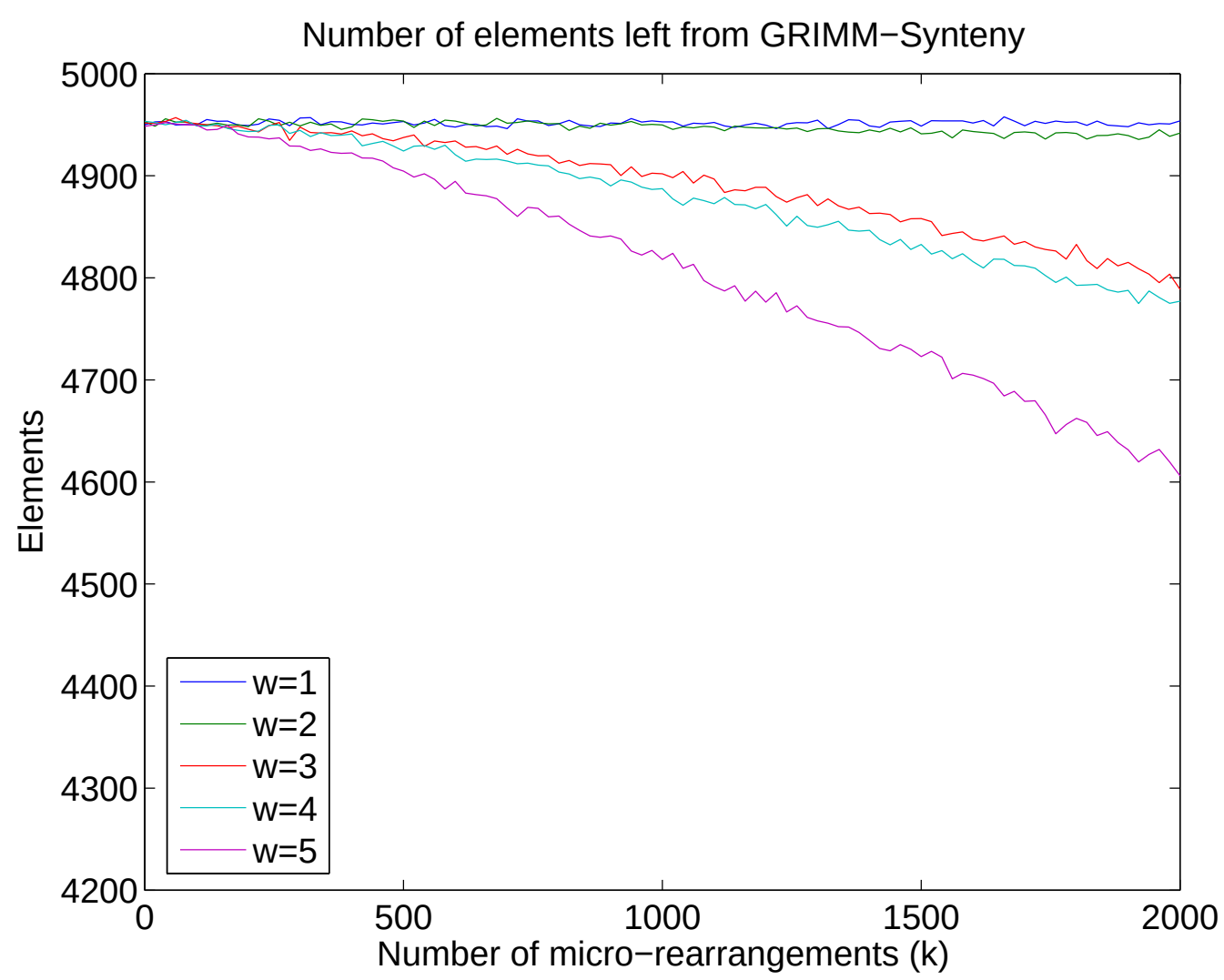

Figure S2. Number of elements retained in simulations after deletion of small blocks. Number of elements retained by (A) ST-Synteny and (B) GRIMM-Synteny, as a function of the number of micro-rearrangements k and micro-rearrangement size w in the simulated data.
